# A Rapid Appraisal of How Alcohol Is Screened and Treated Amongst Minoritised Ethnic Service Users Within Community Mental Health Settings

**DOI:** 10.1111/dar.70118

**Published:** 2026-02-26

**Authors:** Jo‐Anne Puddephatt, Paul Marshall, Duncan Swiffen, Juliana Onwumere, Jayati Das‐Munshi, Ross Coomber, Laura Goodwin

**Affiliations:** ^1^ Department of Psychology Edge Hill University Ormskirk UK; ^2^ Division of Health Research Lancaster University Lancaster UK; ^3^ Mersey Care NHS Foundation Trust Liverpool UK; ^4^ Department of Psychology Institute of Psychiatry, Psychology and Neuroscience, King's College London London UK; ^5^ South London and Maudsley NHS Foundation Trust Michael Rutter Centre London UK; ^6^ NIHR Biomedical Research Centre at South London and Maudsley NHS Foundation Trust and King's College London London UK; ^7^ Institute of Psychiatry, Psychology and Neuroscience, King's College London London UK; ^8^ ESRC Centre for Society and Mental Health King's College London London UK; ^9^ Department of Sociology, Social Policy and Criminology University of Liverpool Liverpool UK

**Keywords:** alcohol, ethnicity, mental health, rapid appraisal, treatment

## Abstract

**Introduction:**

Close to half of those engaged with community mental health teams (CMHT) report an alcohol or drug problem. UK public health guidance recommends that these services screen for harmful alcohol use, but reporting may be less likely amongst minoritised ethnic groups. This study aimed to explore: (i) the prevalence of screening and referrals to alcohol services within CMHTs and differences across ethnic groups; (ii) how alcohol use is assessed and treated in CMHTs, and tailored for minoritised ethnic service users; and (iii) staff and minoritised ethnic service users' experiences of assessing and reporting alcohol use.

**Methods:**

A rapid appraisal was conducted which triangulated data across patient healthcare records (aim 1), online survey (aim 2), interviews and focus groups (aim 3) with three CMHT services within an NHS Mental Health Foundation Trust in North‐West England. Data was analysed using framework analysis.

**Results:**

Both patient notes and survey data showed that alcohol was seldom assessed using formal tools. Three themes were developed reflecting differences in the barriers of reporting and assessing alcohol use for minoritised ethnic service users and staff. With barriers for the former including information sharing and barriers for the latter including protecting the therapeutic relationship.

**Discussion and Conclusions:**

Triangulating data from across different sources highlights the complex challenges that services face in meeting the recommendations around alcohol screening in CMH services. Our findings have implications on the need for staff in mental health services to better understand and accommodate the needs of minoritised ethnic service users who may have co‐occurring alcohol and mental health problems.

## 1 Introduction

In England and Wales, the majority of the population are White British (74.4%), followed by Asian (9.3%), White Other (6.2%), Black (4%) and Mixed (2.9%) [1]. This is also mirrored in the North‐West of England [2]. However, the North‐West has the highest prevalence of common mental health problems (defined as a depressive or anxiety disorder) [3] and a higher incidence of severe mental health problems (defined as bipolar disorder, schizophrenia or other psychotic disorder) [4] compared to other areas in England. It is well established that alcohol and mental health problems commonly co‐occur [5, 6], and longitudinal and mendelian randomisation studies show that worsening mental health increases the consumption of alcohol [7, 8], suggesting that alcohol may be used as a means to cope. Indeed, of those engaged with community mental health (CMH) services, 44% reported a drug or alcohol problem in the previous year [9] though research indicated that there is poor recognition of harmful alcohol use within these services [10].

There are no safe levels of alcohol use [11], and in the United Kingdom (UK), harmful alcohol use is defined as alcohol use which causes adverse health or psychosocial harm [12]. Harmful alcohol use can be assessed using validated screening questionnaires (such as the Alcohol Use Disorder Identification Test or Alcohol, Smoking and Substance Involvement Screening Tool) which assess the frequency of someone's drinking, how much someone drinks on a single occasion and whether they or someone else has been harmed by their drinking. In the UK, alcohol and mental health services are siloed whereby each problem is treated separately with different providers and commissioners. Individuals with an alcohol use disorder (AUD) often struggle to access local mental health services because of this and due to strict referral criteria relating to alcohol use [13]. UK public health guidelines recommend that healthcare professionals in the National Health Service (NHS) should identify harmful drinking and, where needed, recommend a referral to alcohol services for assessment and treatment [14]. More recent guidance also includes further information around substance use and substance use screening and treatment to improve understanding amongst non‐addiction practitioners and encourage screening of substance use in different settings [15]. Guidance also recommended that identification of harmful drinking should be completed through the use of validated screening tools [15, 16]. To date, much of the research on alcohol screening and brief intervention has focused on primary care settings despite statistics showing a high prevalence of harmful drinking amongst individuals receiving support from CMH services [9]. Furthermore, there is an abundance of evidence to indicate that alcohol is not regularly screened in primary care services [17], due to lack of knowledge, training, support from management and fears of harming the practitioner‐service user relationship [17, 18]. There is also limited evidence which has examined how these screening practises may differ amongst specific service user groups who experience additional barriers towards reporting and seeking support for their drinking, such as minoritised ethnic groups.

Coined by Gunaratnam [19], ‘minoritised’ provides a social constructionist approach to explaining that minoritised goes beyond a group being a demographic minority and explains that these groups are actively minoritised by others [20]. In this study, we use the term ‘minoritised ethnic’ based on the intersectionality theory whereby people are actively minoritised in the context of their race, ethnic, religious background as well as access to mental health and alcohol support more broadly. It has been shown that minoritised ethnic groups may be less likely to report or seek support for an AUD due to the way in which people are stigmatised for using alcohol in some communities and religious groups [21] and the lack of trust in the confidentiality of alcohol treatment [22]. This may be compounded by other factors, including previous negative experiences with services, experiences of racism [23], and assumptions made from healthcare professionals around the consumption of alcohol due to the ethnicity of the service user [24]. Previous work has reported inconsistencies in the implementation of these practises across practitioners [18] while also reporting that patients prefer to discuss alcohol related issues with non‐specialist healthcare professionals. This echoes findings elsewhere that minoritised ethnic service users may be open to discussing alcohol concerns with non‐alcohol specialist services [23]. However, it is likely that these findings differ when situated in the context of CMH services with minoritised ethnic service users given that: (i) alcohol use is particularly stigmatised within these groups thus the potential implications reporting may have on the individual and their family; and (ii) the lack of alcohol screening and treatment in mental health services.

Taken together, current evidence indicates that mental health and alcohol problems are common, and that alcohol use should be screened within CMH services [16]. But it is not known how commonly alcohol screening takes place in CMH services, and whether these processes are adapted for minoritised ethnic service users. Examining these issues amongst both staff in CMH services and minoritised ethnic service users will enable researchers, healthcare professionals and policymakers to: (i) understand the extent to which recommendations are being implemented in practise [16]; and (ii) whether additional practises are used to facilitate the needs of those from a minoritised ethnic background. The current study aimed to understand and explore: (i) the prevalence of screening for alcohol use and referrals to alcohol services within CMHTs and differences across ethnic groups; (ii) how alcohol use is assessed and treated in the context of someone's mental health and related treatment, and tailored for minoritised ethnic service users; (iii) experiences of reporting and assessing alcohol use from the perspectives of services, CMH staff and minoritised ethnic service users.

## 2 Methods

This study received ethical approval from the NHS Ethics committee (REC reference: 22/NW/0155) with Lancaster University as its sponsor. The following methods and results are reported in accordance with the consolidated criteria for reporting qualitative research [25].

### 2.1 Participants and Sample Size

Rapid appraisal methods were used to interrogate primary data and patient healthcare records. Rapid appraisal aims to quickly generate information to aid decision‐making and tends to be used in health and social care research [26]. These methods focus on: (i) gaining insider perspective; and (ii) triangulating data from a range of resources and collecting data in an iterative way, while adopting a pragmatic and rapid approach [27]. CMH sites were selected based on sites being in the top three most ethnically diverse areas of Merseyside, UK. In the UK, CMH services provide community level mental health support for adults and older adults. Data and participants were selected through four sources: patient healthcare records (*n* = 2603), online survey (*n* = 16), focus groups (two focus groups, *n* = 18) and interviews (*n* = 11, five service providers and six service users, see Table 1). Recruitment and data collection was facilitated through working with service managers and the North‐West Coast Clinical Research Network. Focus groups were conducted face‐to‐face while interviews took place either online or by telephone.

**TABLE 1:** An overview of the data source, sampling method and inclusion criteria.

Source	Sampling	Inclusion criteria	Data collected	Reimbursement
Community mental health patient records	All minoritised ethnic groups were included if they met the inclusion criteria A random sample of White British service users were used to facilitate comparisons between alcohol recordings and screenings Service users engaged with CMHT's in Mersey Care NHS Foundation Trust since 2016	Service user was currently engaged with one of the three selected Mersey Care NHS Foundation Trust CMH services. There was a recording of an alcohol assessment.	Patient records were obtained in January 2022 from three CMHTs in Mersey Care NHS Foundation Trust by DS. These sites were chosen because they represented areas of greatest ethnic diversity within the Trust. Stratified by ethnicity, patient records (from January 2016 onwards) were used to establish: The drinking status of service users Whether a formal alcohol tool was used Whether a referral to alcohol services was recommended.	
Online survey	Purposively sampled through internal communications, posters, and the North‐West Coast Clinical Research Network.	Staff member within one of the selected CMH services.	See Table S1.	Participants were entered into a £50 Love2Shop voucher prize draw
Focus groups	Purposively sampled through internal communications, posters, and the North‐West Coast Clinical Research Network.	CMH member of staff working at one of the selected CMH services.	Two face‐to‐face focus groups took place in October (*n* = 8) and November 2022 (*n* = 10) at two of the sampled sites.	Participants were reimbursed with a £20 Love2Shop voucher or BACS transfer.
Interviews	Service providers Purposive and snowball sampling. Service users Purposively recruited through the North‐West Coast Clinical Research Network.	Service providers Their role involved in the policymaking and commissioning decisions regarding alcohol and/or mental health service provision, or in the delivery of services in Liverpool. Service users Of non‐White British ethnicity. Either currently drink or previously drank alcohol. Engaged with CMH services in Mersey Care NHS Foundation Trust. Aged 18 or older.	Semi‐structured telephone/online interviews were conducted between November and February 2023. Mean length of interview was 23 min and 43 s.	Participants were reimbursed with a £20 Love2Shop voucher or BACS transfer. All service providers declined payment.

Abbreviations: CMH, community mental health; CMHT, community mental health team; NHS, National Health Service.

### 2.2 Materials and Procedures

See Table 1 for a detailed overview of data collected, the materials and methods used differed depending on the data source. Topic guides for focus groups and interviews were developed by the project team and our patient and participant involvement (PPI) group of minoritised ethnic individuals with lived experience of mental health and alcohol problems. All guides broadly covered how and whether services assessed alcohol use and whether these measures were tailored for minoritised ethnic service users, and experiences of asking or answering questions around alcohol use (see Supporting Information for each topic guide). These were audio‐recorded using a digital Dictaphone with only JP, LG and participants present.

Potential participants were sent a participant information sheet, via email, and after confirming eligibility, sent a consent form to complete. Once the consent form was returned complete, a date and time was arranged for an interview while CMH staff signed up to attend one of two focus groups. Interviews and focus groups were conducted by a White British female postdoctoral researcher (JP) while focus groups were also moderated by a White British female Senior Lecturer (LG). Upon completion of the interview or focus group, participants were debriefed and reimbursed. Participants who completed an interview were sent a copy of their transcript to confirm its accuracy. No relationship was established between the researchers and participants prior to the study, and no repeat interviews were conducted.

### 2.3 Analysis

**FIGURE 1:**
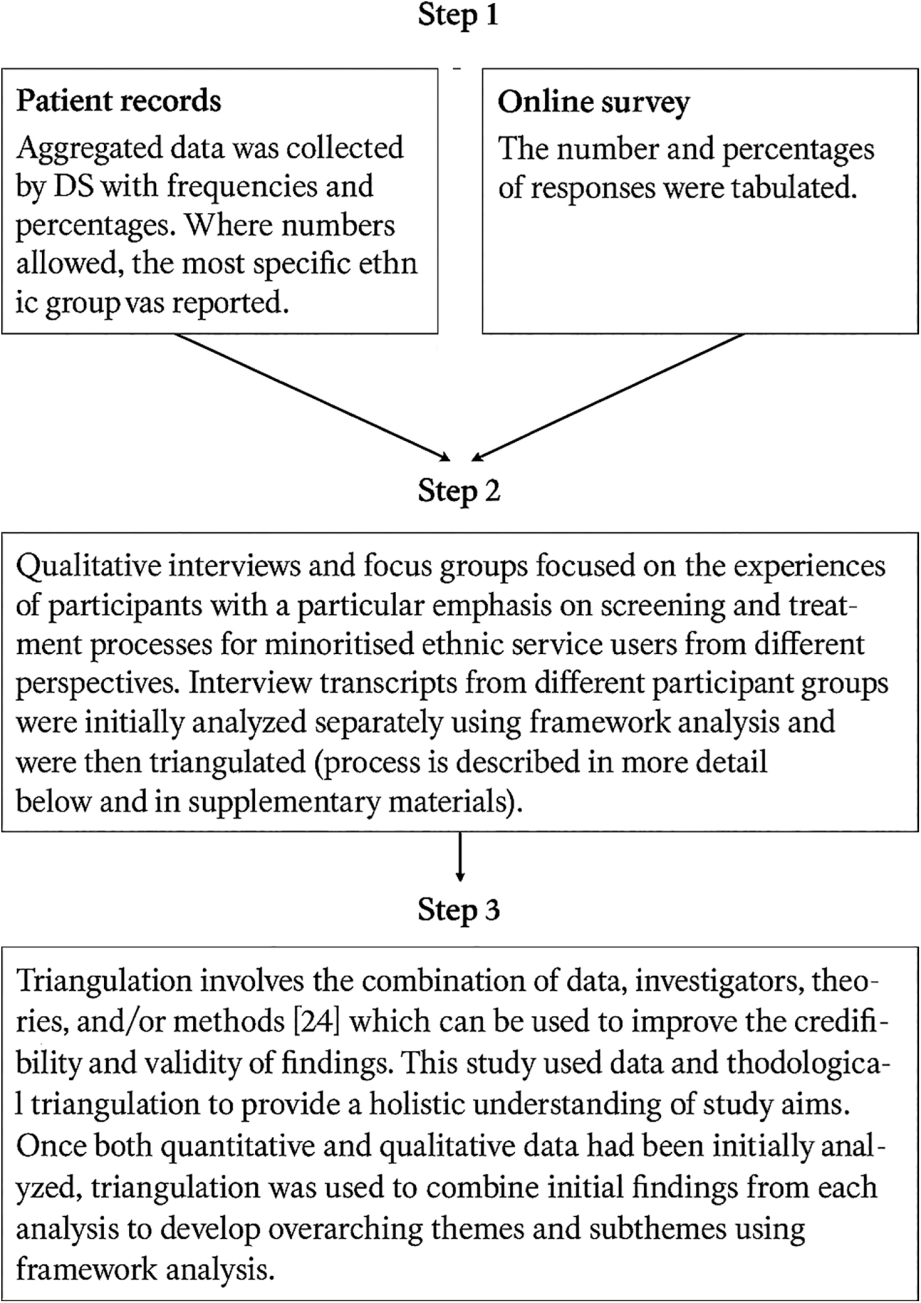
Analytical process.

The analysis took part in three stages (see Figure 1).

Framework analysis is a matrix‐based analytical method which classifies and organises data according to concepts and emerging categories [28], as this study used multiple sources of data, a framework analysis allowed us to identify patterns between different data sources [28, 29]. Framework analysis involves six stages; transcription and familiarisation, initial coding, development of analytical framework, application of analytical framework to all transcripts, charting data into a framework matrix, interpretation. Deductive codes were developed from recommendations on treating people with co‐occurring mental health and drug/alcohol problems and screening people with AUD [14, 30], more recent guidelines were not available at the time of the study commencing [16]. Inductive codes were also developed through familiarisation with interview transcripts.

After re‐reading each transcript, JP inductively and deductively coded four transcripts and developed an initial working analytical framework which was shared and revised through meetings with a second White British male doctoral researcher (PM) who reviewed a proportion of transcripts. JP then applied the analytical framework to all transcripts and charted the data into a framework matrix using Microsoft Excel which provided summaries of each code per transcript (see example in Supporting Information). Data in the matrix was triangulated with patient records and online survey, specifically findings from patient records and survey were mapped onto the corresponding category. Summary reports from NVivo 12 were also used to ensure that developing themes accurately reflected raw data. Developing themes and subthemes were reviewed by PM, DS, project team and PPI group. The analysis was facilitated by field notes and memos (see reflexivity statement in the Supporting Information).

## 3 Results

### 3.1 Patient Records From Selected CMH Sites

There were 2603 service users with a record that referenced alcohol (e.g., refers to the service users drinking status or level of drinking, see Table 2). Mixed ethnic service users had the highest proportion of drinkers (45%) while Asian ethnic service users had the lowest proportion (14%). There were relatively low proportions of ex‐drinkers across ethnic groups (range: 5%–14%). Asian ethnic service users had the highest proportion of non‐drinkers (33%) while Mixed Other ethnic service users had the lowest proportion (7%). Validated alcohol screening questionnaires were rarely used (regardless of ethnicity, range: 0%–8%). The proportion of service users who had been referred to alcohol services was low (0%–19%) though the proportion of referrals was highest amongst White British groups (19%) followed by Mixed Other (14%) and any other ethnic group (14%, data not shown due to small numbers). These records indicate that: (i) alcohol use is not screened for using validated tools for any of the ethnic groups studied; and (ii) White British service users were more likely to receive a referral for alcohol support.

**TABLE 2:** Characteristics of service users.

		Ethnicity							
		White British (*N* = 492/2126)	White other (inc. White Irish) (*N* = 63)	Black African (*N* = 58)	Black Caribbean and Black Other (including Black British) (*N* = 112)	Asian (including Indian, Pakistani, Bangladeshi, Chinese, Asian British) (*N* = 86)	Mixed (White and Black African and White and Black Caribbean) (*N* = 40)	Mixed other (*N* = 54)	Any other ethnic (including Arab) (*N* = 64)
		*N* (%)	*N* (%)	*N* (%)	*N* (%)	*N* (%)	*N* (%)	*N* (%)	*N* (%)
Gender	Male	251 (51.0)	30 (47.6)	32 (55.2)	70 (62.5)	54 (62.8)	24 (60.0)	29 (53.7)	41 (64.0)
	Female	241 (49.0)	33 (52.4)	26 (44.8)	42 (37.5)	32 (37.2)	16 (40.0)	25 (46.3)	23 (36.0)
Age (mean)		48	43	44	48	46	43	42	44
Primary mental health diagnosis	Psychosis	204 (41.5)	14 (22.2)	41 (70.1)	80 (71.4)	41 (47.7)	22 (55.0)	25 (46.3)	36 (56.3)
	Bipolar or Mood disorder	155 (31.5)	23 (36.5)	10 (15.9)	22 (19.6)	21 (24.4)		11 (20.4)	17 (26.6)
	Posttraumatic stress disorder or anxiety disorder	42 (8.5)	10 (15.9)	a	a	9 (10.5)		a	8 (12.5)
	Other (including personality disorder)	89 (18.2)	16 (25.4)	a	9 (8.0)	15 (17.4)	9 (20.0)	12 (22.2)	a
Drinking status (initial assessment)	Drinker (if frequency, consumption or reporting that they drink is recorded)	161 (32.7)	13 (20.6)	37 (25.9)	37 (33.0)	12 (14.0)	18 (45.0)	21 (38.9)	a
	Ex‐drinker	44 (8.9)	9 (14.3)	14 (5.2)	14 (12.5)	a	a	a	a
	Non‐drinker	190 (38.6)	29 (46.0)	23 (39.7)	48 (42.9)	41 (47.7)	15 (37.5)	14 (25.9)	35 (54.7)
	Not known (no alcohol assessments recorded in their notes/no formal alcohol assessment completed)	97 (19.7)	12 (19.0)	17 (29.3)	13 (11.6)	28 (32.6)	a	15 (27.8)	17 (26.6)
Was a validated alcohol screening questionnaire used?	Yes	a	a	a	a	a	a	a	a
	No	486 (98.8)	62 (98.4)	57 (98.3)	107 (95.3)	86 (100.0)	37 (92.5)	33 (98.1)	63 (98.4)
Number of drinkers referred to alcohol services (detox, staff‐referral, self‐referral)		31 (19.3)	a	a	a	a	a	a	a

^a^ Numbers less than 8.

### 3.2 Online Survey

Twenty CMHT staff began the survey, two (10%) did not consent to complete the survey and two (10%) did not complete the survey, therefore, 16 (80%) participants completed the full survey. Participants held a range of professional roles (see Table 3). Most staff (69%) assessed alcohol use through weekly alcohol consumption rather than a validated alcohol screening questionnaire. If a service user drank at hazardous or harmful levels, then participants recommended self‐referral to an NHS drug or alcohol service (38%) but would make a referral to an NHS drug or alcohol service for dependent drinking themselves (38%, regardless of ethnicity). While alcohol screening and referrals within CMH services seemed to be consistently delivered for service users from different ethnic backgrounds, staff felt that the treatment pathways for people with a mental health problem who were drinking at a dependent level were less suitable (75%). Findings from our online survey indicate that the processes in place to assess and treat alcohol use in CMH services were not tailored for minoritised ethnic service users.

**TABLE 3:** Roles of staff who took part in interviews and focus groups.

Source	Professional roles held
Service providers	Service delivery manager (*n* = 2)
	CMH team leader (*n* = 1)
	Community inclusion team leader (*n* = 1)
	Widening participation lead (*n* = 1)
CMH staff	CMH nurse (*n* = 7)
	Homeless outreach worker (*n* = 1)
	Homeless outreach nurse (*n* = 1)
	Nursing associate (*n* = 3)
	Employment specialist (*n* = 1)
	Clinical psychologist (*n* = 1)
	Personal assistant (*n* = 1)
	Doctor (*n* = 2)
	Social worker (*n* = 1)

Abbreviation: CMH, community mental health.

### 3.3 Qualitative Analysis

**FIGURE 2:**
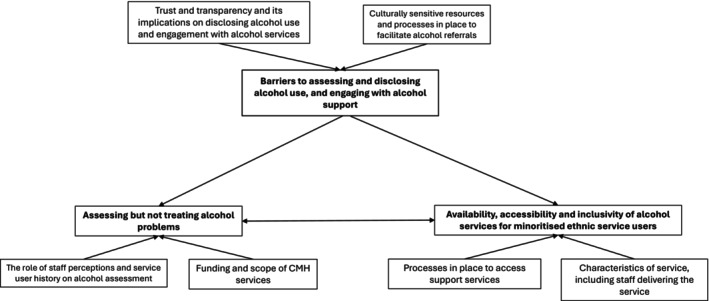
Thematic map of themes and subthemes.

Service providers primarily represented those managing or delivering mental health services though one participant managed drug and alcohol services given that CMH staff would submit referrals to drug and alcohol services (see Table 3). CMH staff represent those in a variety of roles including nursing associates, nurses, psychologists and social workers (see Table 3). Service users represent those currently engaged with community mental health services in Mersey Care NHS Foundation Trust. Three themes were developed which reflected a range of factors which influenced whether alcohol use is assessed or reported, in CMH services, implications of siloed alcohol and mental health services, and accessibility of alcohol services both because of having a mental health problem and being from a minoritised ethnic background (see Figure 2).

#### 3.3.1 Theme One: Factors Influencing Whether Alcohol Is Assessed or Reported

There were a range of issues raised from stakeholders, staff and minoritised ethnic service users which influenced both the way in which alcohol was assessed (if at all)/reported and the outcome of the assessment. Given that the priority of CMH services are around treating mental health problems, if harmful alcohol use is identified within mental health services then this could prevent individuals being treated for their mental health. The assessment and treatment of alcohol disproportionately disadvantaged some minoritised ethnic groups as participants frequently highlighted concerns around alcohol reporting and the impact this may have on their family, housing and immigration status. Underpinning this theme was the importance of the therapeutic relationship for both staff and service users as this was a crucial element which enabled staff to feel comfortable assessing alcohol use and for service users to trust staff when reporting their alcohol use.

Subtheme one: Definition and recognition of alcohol problems and the role of culture.

For minoritised ethnic service users who had previously had an alcohol problem, they described a lack of self‐recognition of their alcohol problem which may stem from their lack of direct experience with other people who were alcohol dependent or where alcohol problems were not commonly seen in some communities. This also corresponded with CMH staff observations though they felt that some of this lack of recognition may have been due to the individuals ability to function.

I didn't identify myself as an alcoholic because I didn't drink every day. I wasn't my, you know, round the clock drinker. So I didn't think I was an alcoholic. (P1, female, mixed, service user)

Sometimes you know it's not all about me. It's like there's other people … in a little bit of a worse state of mind who maybe has to be, had to go first.

Interviewer: And how did you manage, you know, whilst you were waiting to be seen by those services? Respondent: With drugs and alcohol.

(P6, male, Black African, service user)

We see it as alcohol dependency and a significant amount of alcohol to drink, but that person says that's absolutely fine for me, I function on that, but if it goes anything over that then it's a problem. (P8, CMH nurse)

Given that alcohol and alcohol problems may be less visible in some communities, minoritised ethnic service users found peer support groups (whereby personal stories with alcohol are shared) were particularly useful for them to recognise that their drinking may be a problem.

It was a bit like deer in headlights listening to everyone. Because people are talking about the same experiences that you go through, and that's when it comes to realisation. (P1, female, mixed, service user)

Subtheme two: A need for culturally tailored services.

Currently in the UK, alcohol and mental health problems are treated by different services, and people with co‐occurring alcohol and mental health problems often have difficulty accessing support from both services. Accessing alcohol support may be particularly difficult for those with co‐occurring problems from a minoritised ethnic background because of the taboo of drinking alcohol, which may be less well understood in mainstream alcohol services. Such issues with access and engaging with alcohol services have been highlighted amongst CMH staff.

[service users] at least have to be willing to be looking to change that [drinking] in order to access that [specialist mental health] service, and that if the patient is not willing to acknowledge that, then it kind of leaves us stuck for what what's their treatment now with us because we're just managing risk at that point. (P8, CMH nurse)

Across interviews and focus groups, staff highlighted the need for their service to deliver the same care for service users, regardless of their cultural background. However, participants whose service specifically supported people from a minoritised background felt that culturally appropriate, equitable care should be the focus of mental health and alcohol services to better address barriers to assessing alcohol use.

Also is a barrier is cultural awareness about the [mental health] services, some of them they are not aware how to speak with the BME (Black minoritised ethnic), what kind of support they need. They think that they it's like equal, like the White British people, but no, they need extra support. (P45, service provider)

Indeed, CMH staff noted that most of the service users they support were White British while those from a minoritised ethnic background present to services less frequently and perhaps present with different symptoms which may delay them receiving appropriate mental health support. One approach used frequently to support minoritised ethnic service users who do not speak English in CMH settings was the use of interpreters, however, other approaches were seldom used. The reliance on interpreters or family members was a barrier for staff as changes in their reports were observed for fear of information being led back to their community. Illustrating a need to consider other ways to facilitate discussions with minoritised ethnic service users.

[service user] asked the translator to leave the room and we tried to have a conversation, very broken English about it [alcohol] because they had a translator who was from the same community as them and went and disclosed to the community because they mentioned something about alcohol. (P16, doctor)

I think a lot of people who have come from ethnic minoritised backgrounds who have admitted that they drink, but there's also times when we've had family members come into the appointment. and then everything is fine. (P6, CMH nursing associate)

Accounts of services which were culturally adapted outlined work to address some of the structural barriers described above. Specifically, improving links with minoritised ethnic communities, changing inclusion criteria and referral processes for their service. However, these services continued to have lower engagement from minoritised ethnic groups compared with White British groups, indicating that improving links and adapting criteria may not be sufficient in increasing engagement with minoritised ethnic service users. Participants who supported minoritised ethnic groups highlighted that barriers may be addressed through having a link worker who can work with the minoritised ethnic service user to address issues related to access and building trust.

[our service] very much about building that relationship and but then also ensuring that staff feel equipped to work with clients of different ethnicities, different cultures to reduce the risk of those negative experiences, you know, inadvertently being repeated. I guess this is what we found it's [ethnic inequalities] such a big, big area it feels like there are so many different things that we can be doing. (P46, service provider)

Subtheme three: Building and maintaining a therapeutic relationship.

For CMH staff, building a therapeutic relationship, particularly with minoritised ethnic service users, was a priority to facilitate potentially sensitive conversations around alcohol use, with staff highlighting that they often found it difficult to challenge responses to alcohol questions due to the fear of this impacting their relationship with the service user.

if you get to know someone if they're on CPA (care programme approach) level of care, then you're going to see them more regularly. You might start to notice things to then you could sort of question that a little bit more, and also you've built a bit more of a relationship with that person, so it's more comfortable if they are saying ‘no I don't drink’ and then presenting as though they are then you can challenge that a little bit more once you do know them. (P7, CMH nurse and team leader)

For minoritised ethnic service users, there was a lack of trust with mental health services with concerns around how information reported is shared both with other services and to the community. This was particularly problematic for service users who had dependent children. It seemed that some staff were also aware of this lack of trust through their own professional experiences whereby additional resources to facilitate appointments (e.g., use of interpreters) were rejected by service users.

I had a lot of social workers involved in the children's lives as well, so I was dubious to tell doctors what was really going on in case of social services. (P1, female, mixed, service user)

I've had it couple of times where they said they'd rather not have an interpreter … because you know them from their own communities and the worry that it might get around even though confidentiality but there's still fear that it will get back to the community. (P1, stakeholder)

Both staff and stakeholders also identified other groups of minoritised ethnic service users who may be less likely to report any alcohol use, such as asylum seekers or those with precarious residency. Therefore, while building a therapeutic relationship may help to overcome some of these issues to assess and treat alcohol use, there needs to be more explicit discussions with service users about how their information will be used to build trust between service users and staff.

Often my own professional experience with that given people from ethnic backgrounds who have an issue with alcoholism, especially for those seeking asylum in the process of being approved for immigration. They sometimes think that if they admit having substance problems, that's going to have a negative impact on them being allowed into the UK permanently, so they won't say anything because they're fearful. (P10, CMH nurse)

#### 3.3.2 Theme Two: Prioritising Mental Health Needs Within CMH Services

Data from all sources indicated that alcohol was not routinely assessed using validated alcohol screening questionnaires, this largely seemed to be due to mental health services focusing on treating mental health problems only. Though it seemed that alcohol use more regularly assess where service user's had a history of alcohol problems. The latter can be particularly problematic when the service user is from a minoritised ethnic background due to previously discussed issues around alcohol reporting within the context of someone's social, cultural and ethnic background.

Subtheme one: The role of staff perceptions and service user history on alcohol assessment in mental health services.

Current UK public health guidelines recommend that alcohol is routinely screened in healthcare services, including mental health services. Data from patient records indicated that alcohol was rarely assessed using a formal alcohol screening tool though qualitative data suggested that staff did assess alcohol in CMH services but the likelihood of this taking place was influenced by staff's professional roles and the purpose of an appointment. An example in which alcohol was more likely to be assessed was when nursing associates conducted physical health checks which required administration of validated alcohol screening questionnaires. For more senior nursing staff, the use of alcohol assessments was at their discretion and could be delivered in a less structured way. This may explain the lack of uptake of validated alcohol screening tools in both the patient records and online survey.

But if we're just getting onto somebody for an assessment, then it's [alcohol use] something that's usually always touched on … I personally wouldn't go straight out with anything to audit somebody on alcohol and drug use, it would just be an open question like do you use alcohol?. (P7, CMH nurse and team leader)

Regular alcohol assessments within CMH services were also influenced by a service users' previous history with alcohol (regardless of their ethnic background). The below quote is an example of a service user who had previously experienced problems with alcohol which were known to CMH services, therefore, the service user described alcohol assessments routinely taking place.

yeah always … that'll be one of the first questions [being asked if they are drinking] I'll take at the minute you still drinking excessively. (P6, male, Black African, service user)

Relying on having a previous history of alcohol problems may be particularly problematic when supporting a service user whose ethnic, religious or cultural background may prohibit them from discussing any alcohol use. Instead, it may be more appropriate to routinely screen for alcohol use at mental health appointments.

Subtheme two: Funding and scope of CMH services.

Within the UK NHS, there are a range of different services and the scope and way in which mental health and alcohol services are funded can have implications on the expertise of staff. Service providers explained that the focus on mental health support in CMH services can be particularly problematic when screening for alcohol problems as it is out of the scope of services and therefore not a focus during time‐limited appointments.

[services] either funded through mental health grants or funded through addiction grants and … Commission services with different pots of funding … so like our [mental health] service could provide support around drinking alcohol, but that's not our emphasis for permission to do mental health. (P2, service provider)

Given that previous research has established that minoritised ethnic individuals may be less likely to access mental health and alcohol services, some of the existing infrastructure to provide these services may need to facilitate widening access through tailoring access routes and support for individuals' sociocultural background. One example of this was through a service provider who have been reviewing the representation of their service users to that of the local area and meeting with communities and other services to address issues with accessing support.

for clients who are from minoritized ethnic backgrounds a lot of what we [our service] do is around that [widening access] … we've been looking at the demographics of the clients on our caseload and looking at ethnicity in particular and comparing that to Liverpool's demographics to see which communities are underrepresented in our service and we've been working with other organisations, … to try and find out a bit more about what the barriers are to accessing our service. (P46, service provider)

#### 3.3.3 Theme Three: Availability of Culturally Appropriate Support for Minoritised Ethnic Groups Who Have a Comorbid Alcohol and Mental Health Problem

There were a limited range of alcohol services that staff could refer service users on to, of which none were tailored for specific minoritised ethnic groups. Service providers emphasised the implications this could have on willingness to engage, particularly if they had previous negative experiences with alcohol services. This theme emphasises issues around the expectations of current alcohol services which may disproportionately affect minoritised ethnic service users as well as differences in what staff and minoritised ethnic service users felt would encourage engagement from minoritised ethnic service users with alcohol services.

Subtheme one: Accessibility of alcohol referral processes.

Triangulation of data sources indicated that staff recommended self‐referral to drug and alcohol services to reflect willingness to engage. There was another local drug and alcohol service which staff and service providers felt was more accessible but still required a formal referral from either staff or service users. While participants across all sources emphasised the importance of motivation to change alcohol use to engage meaningfully with alcohol services, staff highlighted how this could be problematic for minoritised ethnic services users, those less familiar with the UK healthcare system, with low literacy or with limited access to the internet which may delay the service user completing a self‐referral and being able to engage with follow‐up appointments.

I've found that we've got a lot of service users that can't read or struggle to read or write and struggle with technology they do a little bit of support with making a referral. So I just find [alcohol service] a little bit more accessible but that's just my own personal opinion. (P1, service provider)

For minoritised ethnic service users, there was the perception that CMH staff had less awareness of the wider range of alcohol support, such as peer support groups, which they felt may be more appropriate for minoritised ethnic groups if they did not recognise their drinking as a problem or had limited direct experience of seeing alcohol problems within their community.

I think if they would have known about fellowship. Whether it be NA, AA or whatever fellowship got because they've got no knowledge about it, I probably would have (engaged with alcohol services) (P1, female, mixed, service user)

Subtheme two: Characteristics of professionals in alcohol services.

There were differences between staff and service users around the characteristics of alcohol services and improving engagement with alcohol services. For staff, there was the perception that having alcohol services designed to meet the needs of minoritised ethnic service users or more staff from minoritised ethnic backgrounds could improve engagement because they may be more relatable.

I have briefly done a placement in Building Bridges which was specifically a service for ethnic minoritised and BAME individuals. Interestingly, I think rather than the mainstream services being available for the individuals from ethnic minoritised backgrounds are probably more likely to try and go for more specialist services. (P15, clinical psychologist)

However, minoritised ethnic service users who had engaged with alcohol services preferred services with lived experience or peer support elements as this enabled better identification of their own drinking habits. Some service users also preferred speaking with professionals with lived experience of alcohol problems and who were from a minoritised ethnic background, but this seemed dependent on whether they had previous negative experiences with people from different backgrounds. Interestingly, this preference was not outlined in relation to mental health staff and suggests that the treatment and management of alcohol problems may require different skills and understanding. Nonetheless, the differences in preferences between minoritised ethnic service users and CMH staff and service providers indicates that there is a need for mental health services to have a better understanding of the support required from minoritised ethnic service users which may improve engagement with both CMH and drug and alcohol services.

Some of the counsellors have been there themselves and I find they're the ones you can engage with more because they know what you know what you're talking about. Not to say that the people who come through university and college and as in it's good. But you know, it's all about life experience. (P6, male, Black African, service user).

## 4 Discussion

### 4.1 Statement of Principal Findings

Triangulating data from patient records, staff survey responses, and qualitative data from service providers, CMH staff, and minoritised ethnic service users, our findings are four‐fold. First, there were different barriers and facilitators of reporting alcohol use between participant groups which were influenced by either having a mental health problem or someone's ethnic, religious or demographic background. For minoritised ethnic service users, key barriers included fears of the implications of reporting alcohol use, particularly when other services were involved and difficulties recognising that they had a problem with alcohol because alcohol use and/or problems were seldom prevalent in some communities. For staff and service providers, there was a hesitance to question responses around alcohol use to protect the therapeutic relationship with minoritised ethnic service users, with the reliance on interpreters exacerbating these issues. Second, the focus of addressing mental health problems in CMH services meant that alcohol was not routinely screened during time‐limited appointments as per current UK guidelines. Third, there is a lack of culturally tailored alcohol services within Merseyside which can disproportionately disadvantage those for whom English is not their first language, living in precarious housing or with limited technology literacy. Fourth, there were differences in preferences for the types of alcohol services and characteristics of those delivering the service between participants groups, with a preference for peer groups or being seen by staff with lived experience of alcohol problems amongst minoritised ethnic service users due to the perception that they were able to better relate to people with lived experience.

### 4.2 Strengths and Weaknesses of the Study

This study was the first to triangulate patient records, survey, and qualitative data to explore the implementation of current alcohol screening and treatment guidelines with minoritised ethnic service users within CMHTs. The use of triangulation provided a holistic understanding of the lack of implementation of these guidelines and highlighted the complexity of adhering to recommendations in the context of discussing alcohol use in mental health settings with minoritised ethnic groups. Further, triangulating data from different sources and perspectives has indicated discrepancies between the type of alcohol support those from a minoritised ethnic background living with a mental health problem seek. Second, we purposively sampled minoritised ethnic service users, thus providing an insight into the experiences of traditionally underserved groups when reporting alcohol use and receiving support whiles also managing a mental health problem.

While this study has several strengths, the data included were specific to CMHTs within one NHS Trust; therefore, findings may differ across other NHS Trusts located in more ethnically diverse areas. However, this trust covers three geographical regions in the North‐West of England to a population of 1.4 million people. Individual patient records were manually extracted by one researcher, so data from a smaller random sample of White British service users was extracted. It was not feasible within the timeframe of the study to match the random sample of White British service users to the minoritised ethnic sample based on characteristics such as age and gender. Interviews and focus groups were conducted by White female tertiary educated researchers, which may have impacted responses of participants; however, the researchers offered to speak with potential participants prior to collecting data to address any concerns that they may have had and initial findings were discussed with our PPI group. Online survey responses were conducted by convenience sampling and lacked diversity in the demographic characteristics of participants; however, data was collected in this way to improve response rates with staff working in time‐demanding roles. Finally, due to the scope of the study and the inclusion of a range of methods, participant sources, and the number of participants within each element, it was not possible to explore experiences through the lens of sociodemographic backgrounds of participants.

### 4.3 Strengths and Weaknesses in Relation to Other Studies

Although previous research in primary care settings suggests that screening or brief alcohol interventions is not routinely conducted, research has consistently identified similar barriers and facilitators of screening and treating alcohol use, particularly with regards to practitioner‐service user relationship, access to alcohol services and incentivisation by external policy within these services [17]. This was echoed in an earlier systematic review where the practitioner‐service user relationship was important for facilitating sensitive topics and the need for support from management to facilitate alcohol screening [18]. It was unclear from these studies whether validated alcohol questionnaires were used; however, our findings indicate that screening alcohol was much lower within CMHTs. We also found that the practitioner‐service user relationship was particularly important when supporting those from a minoritised ethnic background due to the way in which alcohol may be perceived within different ethnic, religious or cultural groups. However, screening alcohol use with minoritised ethnic service users in CMH settings can be challenging when there are language barriers or a reliance on interpreters.

Previous research on the perceptions of culturally tailored versus mainstream services amongst young minoritised ethnic groups suggests divided opinion on tailored services with peer support groups (e.g., Alcoholics Anonymous) the most frequently cited alcohol service [31]. This was consistent with our findings; minoritised ethnic service users found peer support groups beneficial in recognising their own alcohol problem through being able to identify with other people's experiences. Yet this was not routinely recommended by staff indicating a disconnect in the perceptions and knowledge of the range of alcohol services available to service users within CMHTs.

To the author's knowledge, the extent to which screening and treatment measures were implemented with minoritised ethnic service users within CMHTs has been seldom explored. One study in health and community‐based settings found that health professionals felt that some aspects of validated alcohol screening questionnaires were helpful but continued to find discussing alcohol with service users difficult [32], however, most studies included in this literature review were not from CMHTs nor focused on minoritised ethnic service users. Another study explored the practise of alcohol brief interventions within a range of health and social care settings and found that staff felt that those with long‐standing mental health problems were not suitable for alcohol interventions [33]. Whilst somewhat consistent with our findings, our study indicates that perceptions of the stigmatising nature of alcohol amongst some ethnic communities, the prioritisation of developing a therapeutic relationship and lack of adapted alcohol services for minoritised ethnic service users exacerbate these issues. Further, these studies did not include the perspectives of service users nor were patient records included to provide an indication of the actual recording of alcohol assessments.

### 4.4 Meaning of the Study

There are several implications of this study for researchers, practitioners, and policymakers. First, our findings have shown a lack of implementation of recommendations regarding the screening of alcohol use using validated alcohol questionnaires with minoritised ethnic groups within CMHTs. Instead, more informal screening takes place but is influenced by a range of factors. For policymakers, there is a need to consider the siloed nature of alcohol and mental health services and how services can be better supported to provide alcohol screening services given the established links between alcohol and mental health, and how processes can be adapted when supporting people from minoritised ethnic backgrounds. For practitioners, a review of available training to screen for alcohol use using validated alcohol screening questionnaires is needed with further cultural training to provide additional support.

Second, there is a need to review the referral processes of alcohol services which disproportionately impact specific groups, including those seeking asylum, those with limited technology abilities, and those in contact with other services (e.g., social services). This study has also shown that both self‐referral and professional referral processes may not be suitable for some minoritised ethnic groups, particularly due to a fear of how information may be used or shared between services and a limited understanding of what alcohol problems look like.

Third, current alcohol services need to consider how they can be adapted for minoritised ethnic service users who may have less understanding of AUDs or concerns with the implications of engaging with alcohol services. This study has shown that service providers and staff referred minoritised ethnic service users to drug and alcohol services, whereas minoritised ethnic service users preferred peer support groups. There is a need for a broader range of alcohol services available to different communities, including the development of services with community outreach to enable culturally adapted alcohol referral processes. Practitioners should work with minoritised ethnic service users to provide information around alcohol use and how this may impact their mental health, as well as discussing the range of alcohol services (including peer support groups).

Fourth, throughout the study we found that there were concerns around the handling of reports of alcohol use and the implications this may have on minoritised ethnic service users. There is a need to review the current procedures in place for supporting minoritised ethnic groups with co‐occurring problems while practitioners should be more transparent with minoritised ethnic service users regarding how their information and responses to questions are managed and shared.

### 4.5 Unanswered Questions and Future Research

While the current study triangulated data from a range of sources, our findings only provide an indication of how alcohol use was screened and treated with minoritised ethnic service users across some CMHTs. Further research is needed to understand the perspectives of service providers and staff from alcohol services given that we found issues with the referral processes put in place by alcohol services. We found that of those who had sought support for their drinking, the use of peer support groups or being supported by services where staff had lived experience with alcohol problems was particularly useful. However, as the current study focused on the identification of alcohol use within CMH services, it was not possible to explore how peer support groups and being supported by people with lived experience of alcohol problems benefitted those with co‐occurring alcohol and mental health problems. This is particularly important because we have shown that those from a minoritised ethnic background may not necessarily engage with drug and alcohol services; therefore, understanding the use of alternative support services and the importance of lived experience may provide some insight into the needs of minoritised ethnic groups with co‐occurring alcohol and mental health problems.

## 5 Conclusions

Using triangulation of primary data and patient healthcare records, this study has established the lack of screening of alcohol use in CMHTs in the North‐West of England and the cultural and structural barriers of reporting and assessing alcohol use within these settings. More support for CMH staff is needed to facilitate the assessment of alcohol use in a culturally sensitive way while culturally adapted alcohol services are also needed to facilitate service users' engagement with alcohol services.

## Author Contributions

Each author certifies that their contribution to this work meets the standards of the International Committee of Medical Journal Editors.

## Funding

This work was supported by Alcohol Change UK.

## Conflicts of Interest

This research was funded under the New Horizons programme by Alcohol Change UK. JD is part supported by the ESRC Centre for Society and Mental Health at King's College London (ESRC Reference: ES/S012567/1) and by the National Institute for Health Research (NIHR) Biomedical Research Centre at South London and Maudsley NHS Foundation Trust and King's College London and the NIHR Applied Research Collaboration South London (NIHR ARC South London) at King's College Hospital NHS Foundation Trust and Population Health Improvement UK. The views expressed are those of the author[s] and not necessarily those of the ESRC, NIHR, the Department of Health and Social Care or King's College London.

## Supporting information


**Data S1:** Supporting Information.

## Data Availability

The data that support the findings of this study are available on request from the corresponding author. The data are not publicly available due to privacy or ethical restrictions.
