# Supplementary material for: A Rapid Appraisal of How Alcohol Is Screened and Treated Amongst Minoritised Ethnic Service Users Within Community Mental Health Settings
Source: Drug Alcohol Rev. 2026 Feb 26;45(3):e70118. doi: 10.1111/dar.70118 (PMC12945475; doi:10.1111/dar.70118)
Supplement: Supplementary file 1 — Data S1: Supporting Information. [file DAR-45-0-s001.docx]

**Supporting Information**

**Table S1: Online survey and responses (*n*=16)**

| **Survey questions** |  | ***n* (%)** |
| --- | --- | --- |
| What is your occupation? | Care Co-ordinator | 1 (6.3) |
|  | Clinical Psychologist | 1 (6.3) |
|  | Community mental health nurse | 5 (31.3) |
|  | CT Doctor | 2 (12.5) |
|  | Psychiatrist | 1 (6.3) |
|  | Support worker | 2 (12.5) |
|  | Team Leader | 2 (12.5) |
|  | Therapist | 1 (6.3) |
|  | Other | 1 (6.3) |
| How long have you worked in that role in that team? | Less than one year | 5 (31.3) |
|  | One to two years | 4 (25.0) |
|  | Three to four years | 3 (18.8) |
|  | Four to five years | 2 (12.5) |
|  | Five to 10 years | 1 (6.3) |
|  | More than 15 years | 1 (6.3) |
| How would you typically identify or find out whether someone is drinking at a problematic level? | Self-reported weekly alcohol consumption | 11 (68.8) |
|  | Use an alcohol screening tool | 3 (18.8) |
|  | I would not ask them and it depends if they want to talk to me about their drinking | 1 (6.3) |
|  | Other | 1 (6.3) |
| How would you typically identify or find out whether someone from a racial or ethnic minority background is drinking at a problematic level? | Self-reported weekly alcohol consumption | 11 (68.8) |
|  | Use an alcohol screening tool | 3 (18.8) |
|  | I would not ask them and it depends if they want to talk to me about their drinking | 1 (6.3) |
|  | Other | 1 (6.3) |
| Which screening tools or questionnaires do you use to identify whether someone is drinking at problematic levels? | Alcohol consumption (amount and frequency) | 5 (31.3) |
|  | AUDIT-C | 3 (18.8) |
|  | AUDIT | 1 (6.3) |
|  | CAGE | 1 (6.3) |
|  | FAST | 1 (6.3) |
|  | None | 5 (31.3) |
| Would you use the same tool or questionnaire if someone was from a racial and ethnic minority background? | Yes | 15 (93.8) |
|  | No | 1 (6.3) |
| What would be the typical action you would take for someone who was drinking hazardously or harmfully (at levels which might be harmful to their health, for example drinking more than 14 units per week but not alcohol dependent)? | Refer to NHS drug and alcohol services | 6 (37.5) |
|  | Suggest a referral to NHS drug and alcohol services | 6 (37.5) |
|  | Provide brief advice | 4 (25.0) |
| Could you tell us which actions you would take if someone from a racial or ethnic minority group was drinking at hazardous or harmful levels? | Refer to drug and alcohol services | 6 (37.5) |
|  | Suggest a referral to drug and alcohol services | 5 (31.3) |
|  | Provide brief advice | 5 (31.3) |
| What would be the typical actions you would take for someone who was drinking dependently (e.g. when someone experiences strong craving and withdrawal symptoms when they are not drinking)? | Refer to NHS detoxification centre | 4 (25.0) |
|  | Refer to drug and alcohol services | 6 (37.5) |
|  | Suggest a self-referral to drug and alcohol services | 4 (25.0) |
|  | Suggest a self-referral to other drug and alcohol services | 2 (12.5) |
| Could you tell us which actions you would take if someone from a racial or ethnic minority group was drinking at dependent levels? | Refer to NHS detoxification centre | 4 (25.0) |
|  | Refer to drug and alcohol services | 6 (37.5) |
|  | Suggest a self-referral to drug and alcohol services | 4 (25.0) |
|  | Suggest a self-referral to other drug and alcohol services | 2 (12.5) |
| Do you think mental health services are as suitable for ethnic minority groups compared to White British groups? | Just as suitable | 7 (43.8) |
|  | More than suitable | 1 (6.3) |
|  | Less than suitable | 8 (50.0) |
| What do you think about the current treatment pathways/options for people with mental health problems who are also drinking at dependent levels? | Are suitable | 3 (18.8) |
|  | Less than suitable | 12 (75.0) |
|  | Are not suitable | 1 (6.3) |
| Are the treatment pathways/options for people with mental health problems drinking at dependent levels suitable for ethnic minority groups? | Just as suitable | 12 (75.0) |
|  | More suitable | 4 (25.0) |
| Is your organisation involved in collaborating or working with other organisations which provide care for someone who has co-occurring alcohol and mental health problems? | Yes | 12 (75.0) |
|  | No | 3 (18.8) |
|  | Not known | 1 (6.3) |
| Do you think these organisations are suitable/culturally appropriate for racial and ethnic minority groups? | Yes | 9 (56.3) |
|  | No | 6 (37.5) |
|  | Not known | 1 (6.3) |
| If someone from an ethnic minority background has an alcohol and mental health problem, is there additional support available within your service to help (e.g. an interpreter if English is not their first language)? | Yes | 14 (87.5) |
|  | No | 2 (12.5) |

**Interview schedules**

Service providers

1. What is your role and what does this involve?
2. What do you think are the current priorities of your organisation?
3. Follow-up: Do you know if any of these priorities are focused around the care for ethnic minority groups?
4. What services are you aware of in the North-West of England which provide services for co-occurring mental health and alcohol problems?
5. Follow up - Who would you say uses these services (for example, males, females, younger people, ethnic groups)
6. Follow up – Can you describe any issues service users who require mental health support but currently drink alcohol at harmful levels may have?
7. In terms of the mental health services available, what considerations if any are taken into account for ethnic minority groups accessing this service?
8. Can you think of any challenges that a service user might face in accessing mental health services if they are from a racial or ethnic minority background?
9. Are you aware of any initiatives or training that is provided to services and staff around the barriers ethnic minority groups may have when accessing and receiving support for their mental health and/or drinking?
10. How do you think current mental health services could be improved to support racial and ethnic minority groups seeking help?
11. Are there any other comments or issues you would like to raise which you think might be relevant?

Concluding statement

How have you found the discussion?

Community mental health staff

1. Thinking about the people who use your service, can you describe the typical people that you see in your service (for example are they mostly female, from a certain age group or ethnicity)
2. Follow up - Can you describe the ethnic groups you typically see at your service?
3. Thinking about the people you see in your service, do you find that many people who are seen have problems with their drinking?
4. Follow-up – do you find that people who have problems with their drinking differs by ethnicity?
5. If yes, in what way?

Screening for alcohol use

1. When someone first comes to your service, would you ask them about their alcohol use?
2. If yes, what types of screening tools or questionnaires do you use to assess their alcohol use?
3. If no, is there anything that stops you from asking them about their alcohol use?
4. Can you think of additional barriers for racial and ethnic minority groups when asked about their drinking?
5. Follow up – For example, can you think of barriers with the professional they are speaking to?
6. Follow up – can you think of cultural barriers they might have when asked about their drinking?
7. If someone comes to your service multiple times, do you ask them about their alcohol use each time?
8. Is there anything that would stop you from asking them about their alcohol use again?
9. Can you give an example of how a service user from an ethnic minority group has presented to your services where you think they might be drinking at harmful levels?

Treating alcohol use

1. If a service user presents to your service and screens for problem drinking, can you describe what the next steps would be?
2. Follow up – Are there additional considerations if are from a racial or ethnic minority background?
3. What are your experiences of treating or referring someone with an alcohol problems who is from a racial or ethnic minority background?
4. Follow up: are there things you do more of/ less of/ differently
5. Can you think of any ways in which a service user’s ethnic background could be taken into consideration when treating an alcohol problem?
6. Prompt – For example, the setting in which the treatment is delivered, e.g. clinic environment?

Support

1. When someone presents to your service, can you tell me about how support outside of your service are assessed?
2. Follow up – Can you tell me about services which provide support for the family in your service?
3. Follow up – How feasible is it to ask about someone’s support network?
4. When someone is discharged from your services, can you tell me about long-term support that is available?
5. Follow up – Can you tell me about how these are culturally appropriate?

Concluding remarks

1. Are there any other issues you would like to raise that we have not covered?

Minority ethnic service users

1. How long have you been using this service?
2. Can you tell me about your experience of making contact this service?
3. Can you tell me what prompted you to seek help for your mental health initially?
4. Follow up – Who has been involved in your care?
5. How would you describe your family’s views of you getting help for your mental health?
6. Can you describe anything which got in the way of you seeking help?

Mental health service use and screening for alcohol use

Thinking about your drinking…

1. If a drinker - How would you describe your drinking?
2. When you first got help from this service, can you remember if the service asked you about your drinking?
3. If yes – can you tell me more about this occasion?
4. If yes – what was it like for you being asked about your drinking?
5. If yes – how open were you about your drinking with staff?
6. When you have been seen by the service, have they asked you again about your drinking?
7. If so, can you tell me a bit more about this?
8. Can you tell me about anything the service could have done to improve your experience of  discussing your alcohol use?

Mental health service use and treatment for alcohol use

1. Have you ever been referred or treated for your drinking?
2. If yes, can you tell me about your experience of this? Who were you treated by?
3. If no, how would you feel about getting support for your drinking?
4. Do you think that the service was suitable for someone from your ethnic group?

*If they have received help for their drinking then ask…*

1. Can you tell me how you felt getting help for your drinking?
2. How would you describe your family’s perceptions of you getting help for your drinking?
3. Can you tell me about anything the service could have done or provided to have made you feel more comfortable in getting help your alcohol use?

Facilitators and barriers to seeking help

1. Thinking about your experience of getting help, can you tell me about your journey to get help?
2. Follow up – can you describe any barriers that you have had in getting support?
3. Thinking about your experience of getting help, can you describe what your ideal care would look like?
4. Would you prefer to be seen by a healthcare profession who is from the same ethnic background to you?
5. Would you prefer to be seen in a particular setting?
6. If they received help for their drinking. Thinking about your experiences with getting help for your drinking, can you describe things which have stopped you from getting help?

Long term recovery

1. Can you describe any services that were recommended to you that provide longer term support, such as any community organisations?
2. How could long term support be made more tailored towards specific groups, for example, for racial and ethnic minority groups?

Concluding remarks

1. Is there anything else you wanted to add that we might not have covered?

**Reflexivity statement**

**Author backgrounds**

The first author is a stage 1 trained Health Psychologist and Lecturer with a background in mental health and addictions. The first author has also previously worked in clinical settings. The first author has completed formal training in conducting qualitative interviews and analysis.

The second author is a postgraduate researcher in Psychology with previous experience in qualitative methods, specifically realist methods.

The third author is a Clinical Research Fellow within an NHS Foundation Trust, has completed core psychiatry training and is a member of the Royal College of Psychiatrists.

The fourth author is a Clinical Psychologist with expertise in psychosis and minority ethnic groups, and qualitative methodologies.

The fifth author is a Clinical Reader and Honorary Consultant Psychiatrist with expertise in severe mental illnesses.

The sixth author is a Professor in Criminology and Sociology with expertise in addictions and conducting qualitative research.

The senior author is a Senior Lecturer in Psychology with expertise in co-occurring alcohol and mental health problems.

**Data collection**

Due to complexity and time demands of the study, the project team worked closely with service managers to identify people who could access patient records and develop a practical study plan for the collection of survey and qualitative data from service providers and community mental health staff. Recruitment was continually reviewed among the project team and worked with the North-West Coast Clinical Research Network to improve recruitment for all participant groups.

*Patient records*

The first, third and senior author developed a search term document to ensure that appropriate patient records were extracted. The third author provided particular expertise on this given their background in Psychiatry and of working within NHS services. The third author searched patient records using the defined search terms and only included records involved with the three specified NHS sites. The team had considered searching all CMHT within one NHS Foundation site, but after a scoping review, it was not feasible to extract all of this data within the timeframe of the study. The first and third author met to compile a table of aggregated patient records which corresponded to the research aims and the first author further group data using broader ethnic categories where cell sizes were small.

*Online survey*

The project team were involved in the development of the online survey which was designed with current guidelines around the screening of alcohol use. There was particular consideration regarding the wording of questions as the team were aware that some staff may not be familiar with current PHE and NICE guidelines regarding the treatment of service users with co-occurring problems. The project team worked with the North-West Coast Clinical Research Network to develop recruitment strategies to enhance uptake. Recruitment was continually reviewed with the team considering alternative strategies.

*Qualitative data*

The project team worked with a Consultant Psychiatrist initially to identify appropriate CMHTs given that the scope of the research was the screening and treatment of alcohol use with minority ethnic service users. The Consultant Psychiatrist put the project team in contact with appropriate service managers and team leaders to initiate discussions around the study plans. The first and senior authors had meetings with CMHTs prior to seeking NHS ethical approval to discuss the feasibility of the research and its methodology. Feedback from these meetings were used to inform the design of the qualitative interviews and focus groups, specifically the mode of data collection and where and when it could take place. Once NHS ethical approval was obtained, the first and senior authors maintained regular contact with some CMHT team leaders, assistant psychologists and the North-West Coast Clinical Research Network to review recruitment.

Interview schedules were initially developed by the first and senior author based on feedback from the participatory involvement group. Interview schedules were then reviewed by the wider project team, participatory involvement group and project advisory group. Revisions were made to these schedules based on feedback. The interview schedules were finalized once approved by the wider project team, participatory involvement group and project advisory group.

**Data analysis**

*Patient records*

The first, third and senior author were involved in the analysis of patient records. The third author’s background provided the team with a unique insight into the interpretation of the data, particularly around the use of formal alcohol screening tools.

*Online survey*

The first author was involved in the analysis of online survey data.

*Qualitative data*

The first author conducted all interviews and focus groups, while focus groups were also moderated by the senior author who took notes during the focus group. The first author transcribed and anonymised all transcripts, and these were checked for accuracy using the digital recording of the interview/focus group. The second author reviewed a proportion of anonymised transcripts. The first author developed an initial analytical framework based on reviewing a proportion of transcripts as well as guidance and recommendations for screening and treating alcohol use [1, 2]. This was reviewed by the second author and revisions were made. Both the first and second author coded the same proportion of transcripts using the initial analytical framework to establish reliability. Once established, the first and second author met to discuss the framework which was reviewed by the wider project team before final revisions were made.

The first author coded all transcripts using the analytical framework and developed a preliminary matrix where similar codes were combined which was then reviewed and discussed with the project team. Once the matrix was refined, the first author provided summaries based on raw participant data per category and developed an initial set of themes and subthemes for each participant group which was discussed among the project team and participatory involvement group.

**Comparison of data within and across cases in the dataset**

Transcripts and coding were managed in NVivo while the analytical framework was facilitated through a matrix in Excel. Rows reflected individual participants while columns reflected participant characteristics, participant group, as well as broader categories. Comparing data within cases allowed the first author to explore contextual meaning which was particularly for this study. During the latter stages of analysis, cases were also compared across the qualitative dataset which facilitated the search for similarities and differences between participant groups. Finally, the first author also compared the qualitative data to the main findings from patient records and online survey.

**Use of memos and field notes**

The first author made notes of each interview while the senior author made notes of each focus group, including observations and interesting topics that may warrant further exploration. These notes were used to facilitate the initial stages of analysis but to also assist in the triangulation across all sources of data. The first author also used memos throughout the analysis to provide an audit trail as the analysis progressed from raw data to interpretation to finalization of themes.

**Triangulation**

Data were analysed individually and then triangulated towards the final stages of the analysis, therefore, memos were crucial in developing and finalizing themes and subthemes. Patient records and online survey data provided the project team with an understanding of the extent to which alcohol data was recorded and the extent to which alcohol problems are treated with CMHTs. Qualitative data provided the project team with a better understanding of findings from patient records and online survey data and these were constantly reviewed in the development of themes and subthemes.

**Prior assumptions and experience**

The current study was part of a larger project which had previously established the associations between alcohol and mental health across ethnic groups in England, and the experiences with alcohol among minority ethnic individuals with a mental health diagnosis. The findings from the previous work led to the development of the current study as the team identified issues regarding the recognition of alcohol problems and accessing formal services but there was seldom research in this area within the context of CMHTs.

**Interview setting and relationship between the researcher and participant**

Interviews and focus groups took place either by telephone, online or face-to-face. This decision was made after several discussions among the project team and with CMHT team leaders and the North-West Coast Clinical Research Network. While the first and senior author had established a relationship with CMHTs, no prior relationship between the researcher and those taking part was established prior to collecting data. All participants were encouraged to ask questions about the research prior to consenting to take part in the study. The first author was conscious that some members of staff may be concerned about disclosing some information due to the focus group setting. The first author outlined ground rules, including the need to keep responses within the focus group and reiterated that data would be anonymised, to overcome any potential concerns.

**Table S2: Example of framework matrix**

| **Participant ID** | **Source** | **Professional role** | **Alcohol services available to minority ethnic groups screening for problem drinking** | **Experiences of answering questions about their drinking** | **Identification of alcohol use** | **Priorities of individuals, staff and organisations** | **Problems with using additional resources to support minority ethnic groups** | **Referring people to other services** | **Take into account that stigma and discrimination are associated with alcohol misuse** |
| --- | --- | --- | --- | --- | --- | --- | --- | --- | --- |
|  | Patient records |  | Patient records indicate that HCPs refer to detox or drug and alcohol services. A large proportion of recordings indicate that self-referral is recommended but it is not possible to clarify whether a referral was made. |  | While the data included refers to service users where there is mention of alcohol, there are differences in the proportions of current and non-drinkers across ethnic groups. However, the data suggests that the majority of service users’ alcohol use is not assessed using formal alcohol screening tools. |  |  | While it was not possible to establish whether referrals to alcohol services were made, data suggests that recommendations are usually made to formal alcohol services rather than non-statutory services, e.g. AA. However, there seems to be a higher proportion of recommendations towards self-referral rather than staff-referral. |  |
|  | Online survey | Participants who completed the survey held a range of roles from junior level (e.g. support worker) to senior level (e.g. psychiatrist). The majority of participants had only recently been in these roles (< two years). ***It is useful to have gained responses from a range of professional roles because they may have different interactions with minority ethnic service users.*** | The majority of participants recommended referring minority ethnic service users to formal alcohol services, though the specific alcohol service and how the referral would be made seemed to depend on the severity of alcohol use (e.g. more participants recommended detox or making a referral if drinking dependently). There were some indications of recommending other drug and alcohol services to minority ethnic service users (6.3-12.5%) but it is not clear why this is. ***This corresponds with some of the patient data regarding formal alcohol services and that there may be some differences if the service user is from a minority ethnic group.*** |  | The majority of participants used self-reported weekly alcohol consumption to assess alcohol use (68.8%) compared with an alcohol screening tool (18.8%) which is consistent with patient records. However, it seems that screening for alcohol use is conducted in a similar way with minority ethnic groups. ***This is not consistent with the most recent recommendations regarding the use of formal alcohol screening tools.*** |  |  | Recommended referrals seemed to depend on the severity of drinking where there was an increase of responses towards detoxification if the service user was drinking at dependent levels. There also seemed to be some change in responses if service users were from a minority ethnic background. ***This is consistent with recommended guidelines where detoxification should be recommended if someone is drinking at dependent levels.*** |  |
| P5 | Service provider | Clinical services manager overseeing multiple mental health and addiction services within Mersey Care NHS Foundation Trust. Their focus is on the operations of services and delivering them to high standards as well as overseeing strategic plans | Their role involves overseeing LCAS and Addiction services in Mersey Care NHS Foundation Trust which involves a combination of detox and psychological support for service users with co-occurring problems. The participant has a good awareness of formal alcohol services but refers less to non-statutory services. ***This participant provides a unique insight to alcohol services due to their professional role but acknowledges that alcohol services engage less well with minority ethnic groups due to the way in which it is stigmatized within these groups.*** |  | The participant described the use of formal alcohol screening tools but explained that much of the initial information will have been collected from services who have made the referral. The participant provided limited additional information around this topic but explained the importance of having staff who can work closely in the community with service users and who have lived experience with alcohol problems. ***Some of the limited insight into the identification of alcohol use may be due to the professional role of the participant and speaking from the perspective of alcohol services where some of the initial assessments seem to have already been completed. Nonetheless, their data suggests the need to work within the community that they serve when supporting people with alcohol problems.*** | The overarching focus of the Trust was based around building better relationships between alcohol and mental health services as well as with community and voluntary organisations but the way in which they were improving these links is unclear. ***There seemed to be an acknowledgement of the need for better linking between alcohol and mental health services within a Trust but there are indications that it is not known how to do this effectively which may explain some of the lack of implementation of recommendations?*** |  | The participant described better linking in the referral process between GP practices with drug and alcohol services, with a focus on developing a specific alcohol role to help triage service users. ***The development of roles and improving links with primary care suggests that link workers may become important for drug and alcohol services but it is not clear whether a similar role is being developed within CMHTs, and suggests a focus on primary rather than community services.*** | The participant felt that their was stigma associated with both alcohol and mental health problems but that a central issue of alcohol problems was the limited recognition of when alcohol becomes a problem. ***There was limited discussion of this within the context of minority ethnic service users which may indicate a lack of understanding of issues that minority ethnic groups experience.*** |
| P1 | Community mental health staff | Community mental health nurse |  |  | The participant broadly uses AUDIT questions when they suspect a service user requires a referral to an alcohol service but this seems to be used on an ad-hoc basis rather than routinely assessed and to fulfil the referral criteria of the alcohol service. ***The participant seemed to be selective of who they screen on the basis of need and requirement from alcohol services. The emphasis on conducting this on an ad-hoc basis suggests that alcohol use may not be a priority for the participant which may be reflective of how service users present to services.*** |  | The participant discussed the differences between consultation when they are conducted face-to-face vs telephone as they felt assessments were more difficult when conducted by telephone, particularly when there were issues with dialects. However, they felt that telephone appointments were better with some minority ethnic service users when there was a need to assess alcohol use because they felt the service user was more open. ***This may be important given that these CMHTs have been moved away from residential areas so telephone appointments may be more common but it also suggests that communication may be more difficult because they are reliant on the voice rather than other cues.*** | The participant tends to conduct a more formal screening of alcohol use because this is a requirement of placing referrals to alcohol services. ***This echoes responses earlier in their transcript where formal alcohol tools need to be a requirement for them to be used. The participant also refers to formal alcohol services rather than non-statutory services, indicating that they use these services less.*** | The participant had an awareness of the differences in the acceptability of alcohol across difference cultures and how supportive families may be with these issues. ***This may explain some of the issues raised around conducting telephone and face-to-face assessments of alcohol use.*** |
| P6 | Service user |  | The participant has been engaged with Alcoholics Anonymous but found that this service was mentioned less compared to other formal alcohol services by community mental health staff | The participant described feeling comfortable in disclosing their alcohol use, including past issues with alcohol. This seemed to be underpinned by their motivation to get appropriate support. However, they found discussing their alcohol use when engaged with alcohol support services more beneficial when seen by someone with lived experience of alcohol problems because they felt better understood. ***The participants response suggests that discussing alcohol use is not as much of a problem compared to what staff and service providers believe but that this identification and support may be more helpful to those with alcohol problems if they have experience of it themselves.*** | The participant described being asked about their drinking in terms of more generic questions such as “do you still drink alcohol” at each consultation with the CMHT. Though this does not seem to be problematic for the participant. ***This suggests that alcohol use is routinely assessed for some minority ethnic groups, and combined with data from the focus groups, this may be because of their history of alcohol problems?*** | The participant was empathetic towards CMHTs and alcohol services because of the current landscape of staffing and funding. For the participant, they felt it was important to have regular face-to-face appointments with CMHTs because they prefer to speak to someone that they can see. ***While some staff acknowledge the benefits of telephone appointments, for this participant this was not preferred and suggests that they prefer the communication take place in a face-to-face consultation.*** |  | The participant described good experiences with referrals to CMHTs from the GP though they seem to have a particularly good relationship with their GP. The participant was referred to formal alcohol services through CMHTs but found some staff within alcohol services were less understanding of the journey of recovery from alcohol problems due to their lack of lived experience and instead sought support from AA through their own networks. ***This participants experiences indicate that referral processes can be good and useful when staff do this on their behalf, however, it seems that they are seeking certain characteristics when engaging with alcohol support.*** | While not linked with alcohol use, the participant had early experiences of trauma and racism from their adopted family which has had a marked impact on how they interact with White British people. But the participant seems to be aware of this and feels that this is something they need to resolve rather than can be addressed by being seen by staff from certain demographic backgrounds. ***It is unclear whether their experiences of racism and trauma were related to their alcohol problems but the participant was reluctant to discuss this topic in more detail.*** |

**Additional references**

1. Public Health England. Better care for people with co-occurring mental health and alcohol/drug use conditions: A guide for commissioners and service providers. 2017.

2. National Institute for Health and Care Excellence. Alcohol-use disorders: diagnosis, assessment and management of harmful drinking (high-risk drinking) and alcohol dependence 2011.
